# Spike firing attenuation of serotonin neurons in learned helplessness rats is reversed by ketamine

**DOI:** 10.1093/braincomms/fcab285

**Published:** 2021-12-01

**Authors:** Kouichi Hashimoto, Yosuke Yamawaki, Kenji Yamaoka, Takayuki Yoshida, Kana Okada, Wanqin Tan, Miwako Yamasaki, Yoshiko Matsumoto-Makidono, Reika Kubo, Hisako Nakayama, Tsutomu Kataoka, Takashi Kanematsu, Masahiko Watanabe, Yasumasa Okamoto, Shigeru Morinobu, Hidenori Aizawa, Shigeto Yamawaki

**Affiliations:** 1 Department of Neurophysiology, Graduate School of Biomedical and Health Sciences, Hiroshima University, Hiroshima 734-8551, Japan; 2 Department of Cellular and Molecular Pharmacology, Graduate School of Biomedical and Health Sciences, Hiroshima University, Hiroshima 734-8551, Japan; 3 Department of Neurobiology, Graduate School of Biomedical and Health Sciences, Hiroshima University, Hiroshima 734-8551, Japan; 4 Department of Anatomy, Faculty of Medicine, Hokkaido University, Sapporo 060-8638, Japan; 5 Department of Psychiatry and Neurosciences, Graduate School of Biomedical and Health Sciences, Hiroshima University, Hiroshima 734-8551, Japan

**Keywords:** Kv1 voltage-dependent K^+^ channel, dorsal raphe nucleus, dendrotoxin-I, learned helplessness, 5-HT neuron

## Abstract

Animals suffering from uncontrollable stress sometimes show low effort to escape stress (learned helplessness). Changes in serotonin (5-hydroxytryptamine) signalling are thought to underlie this behaviour. Although the release of 5-hydroxytryptamine is triggered by the action potential firing of dorsal raphe nuclei 5-hydroxytryptamine neurons, the electrophysiological changes induced by uncontrollable stress are largely unclear. Herein, we examined electrophysiological differences among 5-hydroxytryptamine neurons in naïve rats, learned helplessness rats and rats resistant to inescapable stress (non-learned helplessness). Five-week-old male Sprague Dawley rats were exposed to inescapable foot shocks. After an avoidance test session, rats were classified as learned helplessness or non-learned helplessness. Activity-dependent 5-hydroxytryptamine release induced by the administration of high-potassium solution was slower in free-moving learned helplessness rats. Subthreshold electrophysiological properties of 5-hydroxytryptamine neurons were identical among the three rat groups, but the depolarization-induced spike firing was significantly attenuated in learned helplessness rats. To clarify the underlying mechanisms, potassium (K^+^) channels regulating the spike firing were initially examined using naïve rats. K^+^ channels sensitive to 500 μM tetraethylammonium caused rapid repolarization of the action potential and the small conductance calcium-activated K^+^ channels produced afterhyperpolarization. Additionally, dendrotoxin-I, a blocker of Kv1.1 (encoded by *Kcna1*), Kv1.2 (encoded by *Kcna2*) and Kv1.6 (encoded by *Kcna6*) voltage-dependent K^+^ channels, weakly enhanced the spike firing frequency during depolarizing current injections without changes in individual spike waveforms in naïve rats. We found that dendrotoxin-I significantly enhanced the spike firing of 5-hydroxytryptamine neurons in learned helplessness rats. Consequently, the difference in spike firing among the three rat groups was abolished in the presence of dendrotoxin-I. These results suggest that the upregulation of dendrotoxin-I-sensitive Kv1 channels underlies the firing attenuation of 5-hydroxytryptamine neurons in learned helplessness rats. We also found that the antidepressant ketamine facilitated the spike firing of 5-hydroxytryptamine neurons and abolished the firing difference between learned helplessness and non-learned helplessness by suppressing dendrotoxin-I-sensitive Kv1 channels. The dendrotoxin-I-sensitive Kv1 channel may be a potential target for developing drugs to control activity of 5-hydroxytryptamine neurons.

## Teaser

Hashimoto et al. reported electrophysiological changes induced by learned helplessness in dorsal raphe serotonin neurons. The depolarization-induced spike firing was attenuated in rats showing learned helplessness. The firing attenuation was caused by upregulation of dendrotoxin-I-sensitive Kv1 channels. The firing attenuation was reversed by ketamine.

## Graphical Abstract

**Graphical Abstract:**
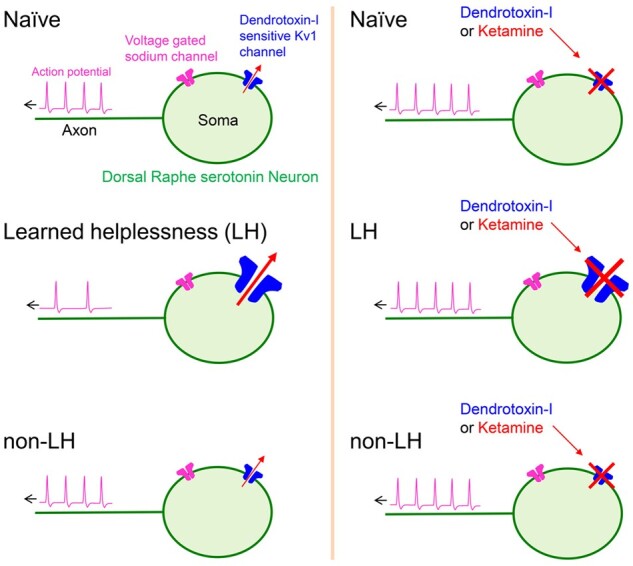

## Introduction

Aversive information is processed in neuronal circuits including the periaqueductal grey, locus coeruleus, dorsal raphe, habenula, amygdala and prefrontal cortex.^1–3^ Chronic pain, stress and anxiety are thought to trigger modulation of these circuits and cause depression- and anxiety-like behaviours. For example, animals suffering from sustained uncontrollable stress often show anxiety-like behaviours and low effort to escape the stress. This behavioural change is called ‘learned helplessness’ (LH) and has been proposed as an animal model useful for the study of depression.^1–4^

Stress-induced activation and modulation of neuronal circuits have been intensively studied in the dorsal raphe nuclei (DRN), one of the major sources of 5-hydroxytryptamine (5-HT) in the central nervous system. Inescapable stress causes hyperactivation of 5-HT neurons in the DRN^5^^,^^6^ and increases the 5-HT level in the DRN^7^^,^^8^ and other brain regions.^9–12^ Lesion of the DRN or pharmacological blockade of 5-HT neurons suppresses LH,^2^^,^^13^ suggesting that activation of the DRN is crucial for inducing depressive behaviours. Hyperactivation sensitizes 5-HT neurons,^9–11^ making 5-HT neurons respond to mild stresses. These lines of evidence raise the possibility that exposure to inescapable stresses changes the electrical activity of 5-HT neurons in LH animals. However, changes in the electrophysiological properties associated with depression-like behaviour in DRN 5-HT neurons are largely unknown.

In this study, we examined the electrophysiological differences of 5-HT neurons in the ventral part of the DRN in naïve rats, LH rats and rats resilient to inescapable shocks (ISs) (non-LH). We found that spike firing was significantly attenuated in LH rats compared with the levels in naïve and non-LH rats. This change was caused by the enhancement of the activity of dendrotoxin-I (DTX-I)-sensitive Kv1 voltage-dependent K^+^ channels. We also found that the antidepressant ketamine reversed the spike firing attenuation in LH rats by suppressing DTX-I-sensitive Kv1 channels.

## Materials and methods

### Rats

Male Sprague Dawley rats aged 4 weeks were purchased from Charles River Japan (Yokohama, Japan). The rats were housed in groups of three in standard polycarbonate home cages (38 × 23 × 20 cm; SEOBiT, Tokyo, Japan). All rats were provided with water and food *ad libitum* and were maintained under specific pathogen-free conditions on a 12-h light/dark cycle (lights on at 08:00 a.m.) at a constant room temperature (23 ± 2°C) and humidity (60%). All animal procedures were conducted in accordance with the Hiroshima University and Hokkaido University Animal Care and Use Committee Guiding Principles on Animal Experimentation in Research Facilities for Laboratory Animal Science (#A20-146–3), and in accordance with the Guidelines for Proper Conduct of Animal Experiments of the Science Council of Japan.

### Learned helplessness

Rats were maintained for 7–10 days in their home cages. The IS was performed as previously described^14^ at postnatal Week 5. The test consisted of an IS session on Day 1 and an avoidance test session (AT session) on Day 2. The experimental room lighting was dim and the rats were habituated to the room for at least 20 min prior to each session. The test apparatus consisted of an experimental chamber (50 × 28 × 32.5 cm) with a stainless-steel grid floor connected to a shock generator-scrambler (SGS-003; Muromachi, Tokyo, Japan). During the AT session, a lever was mounted 5 cm above the grid floor on one wall of each chamber. In the IS session on Day 1, each rat was placed in the chamber and exposed to 80 inescapable foot shocks from the electrified grid floor (single shock intensity: 0.8 mA, shock duration: 15 s, without a light signal, interval: 10–20 s, total time: 40 min). After the IS session, rats were returned to their home cages. The AT session was performed 24 h later, on Day 2. In the AT session, each rat was placed in the same chamber as during the IS session and exposed to 15 foot-shock trials (single shock intensity: 0.8 mA, shock duration: 60 s, trial interval: 24 s). The current was accompanied by a light signal placed above the lever as a clue for detecting the lever and for discriminating the AT session from the IS session.^14^^,^^15^ Each foot-shock trial could be terminated by pressing the lever. If the escape latency, from foot shock to lever press, was <20 s, the trial was considered a ‘success’. If the escape latency was 20–60 s, the trial was considered a ‘failure’. Escape latencies were recorded automatically. Rats with more than 11 failures were classified as ‘LH’, and rats with fewer than 4 failures were classified as ‘non-LH’. Rats with 5–10 failures were classified as ‘intermediate’. This procedure induced an LH status in ∼15–20% of conditioned rats. All of the following experiments were performed until 6 days after the IS session.

### Microdialysis

LH and non-LH rats were anaesthetized with a mixture of medetomidine hydrochloride (0.375 mg/kg, Domitor; Nippon Zenyaku Kogyo Co., Ltd, Fukushima, Japan), midazolam (2 mg/kg, Sando; Sando Co., Ltd, Aichi, Japan) and butorphanol (2.5 mg/kg, Vetorphale; Meiji Seika Pharma Co., Ltd, Tokyo, Japan) dissolved in saline. The guide cannula (AG-12; Eicom, Kyoto, Japan) with a dummy cannula (AD-12; Eicom) was stereotaxically implanted into the DRN. The anteroposterior, mediolateral and dorsoventral coordinates (mm) from the bregma and dura were −6.8, 3.5 and −5.8, respectively. The cannula was lowered at 30° lateral to the vertical plane. The guide cannula was fixed in place with two screws on the skull and dental resin (UNIFAST Trad; GC, Tokyo, Japan). After implantation, the rats were intraperitoneally injected with atipamezole (0.75 mg/kg, Antisedan; Nippon Zenyaku Kogyo Co., Ltd, Fukushima, Japan) as an antagonist of medetomidine hydrochloride.

After a recovery period of at least 2 days, the rats were briefly anaesthetized with ∼2% isoflurane in oxygen and a microdialysis probe (A-I-12–1; Eicom) with a 1-mm artificial cellulose cuprophan membrane (50 kDa molecular weight cut-off) was inserted into the guide cannula. The probe was continuously perfused with Ringer’s solution (147 mM Na^+^, 4.0 mM K^+^, 2.3 mM Ca^2+^ and 155.6 mM Cl^−^) at 1 μl/min in freely moving rats. After a 1-h stabilization period, 10 samples were collected every 5 min to evaluate the baseline 5-HT levels. Then, the probe was perfused with high-potassium Ringer’s solution (high-K^+^ solution; 51 mM Na^+^, 100 mM K^+^, 2.3 mM Ca^2+^, 155.6 mM Cl^–^), and another 10 samples were collected every 5 min. The 5-HT levels were measured by a high-performance liquid chromatography electrochemical detector (HTEC-500; Eicom).

After the microdialysis testing, the animals were deeply anaesthetized with a mixture of medetomidine hydrochloride (1.5 mg/kg), midazolam (8 mg/kg) and butorphanol (10 mg/kg) and sacrificed. Coronal sections (30 μm thick) were prepared for histological analysis to evaluate the DRN injection sites.

### Electrophysiology

At 1–5 days after conditioning, the rats were placed in the chamber and then the CO_2_ level was increased. After losing consciousness, the rats were decapitated. Coronal midbrain slices (300 µm thick) were prepared with a vibratome slicer (VT1200S; Leica Biosystems, Germany) in chilled cutting solution composed of (in mM): 120 choline-Cl, 2 KCl, 2 CaCl_2_, 6 MgCl_2_, 28 NaHCO_3_, 1.25 NaH_2_PO_4_ and 20 glucose bubbled with 95% O_2_ and 5% CO_2_. For recovery, slices were incubated for 1 h in a normal artificial cerebrospinal fluid (ACSF) composed of (in mM): 125 NaCl, 2.5 KCl, 2 CaCl_2_, 1 MgSO_4_, 1.25 NaH_2_PO_4_, 26 NaHCO_3_ and 20 glucose, bubbled with 95% O_2_ and 5% CO_2_ at room temperature.

Whole-cell recordings were performed from somata of 5-HT neurons in the ventro-medial part of the DRN above the decussation of the superior cerebellar peduncle in acute slices (∼−7.7 mm from the bregma)^16^ using an upright microscope (BX50WI; Olympus, Tokyo, Japan). All experiments were carried out at 31°C. The intracellular solution was composed of (in mM): 125 K-methylsulfate, 10 KCl, 5 NaCl, 10 HEPES, 0.5 EGTA, 4 Mg-ATP and 0.4 GTP (pH 7.3, adjusted with KOH). The standard bath solution was the same as the ACSF, and ion channel blockers were added to the ACSF. Spike firing tended to change with time after establishment of the whole-cell recording, probably because of exchange of the intracellular fluid. To minimize the influence of this, data with and without blockers were sampled from different neurons. To sample data with blockers, whole-cell recordings were performed after control ACSF had been switched to that with blockers. Voltage responses were recorded with an Axopatch-1D (Molecular Devices, USA) or EPC-10 (HEKA, Reutlingen, Germany) patch clamp amplifier. The signals were filtered at 3 kHz and digitized at 20 kHz. Online data acquisition and offline data analysis were performed using PULSE or PATCHMASTER software (HEKA).

We identified 5-HT neurons using the following criteria based on their electrophysiological properties:^17–19^ wide half width of action potentials [>0.8 ms, duration at half peak amplitude measured from the baseline membrane potential (≈ −60 mV)], slow time constants (τ > 20 ms) of voltage responses to injection of a hyperpolarizing current of 100 pA, no hyperpolarization-activated potentials and no rebound depolarizations after the offset of hyperpolarizing currents. Most neurons that satisfy these criteria are 5-HT neurons,^18^^,^^19^ with the exception of a very minor population of non-5-HT neurons with similar electrophysiological properties.^17^ If the electrode potential polarization at the end of the recording was over ±5 mV from the initial value, the recording was omitted from the analysis. Furthermore, recordings with high series resistances (over 15 MΩ) were also omitted. Recordings that required holding currents greater than ±30 pA to hold at −60 mV were also omitted.

### Fluorescent *in situ* hybridization

We used non-isotopic *in situ* hybridization with fluorescein-labelled cRNA probes for *Kcna1* (nucleotides 2757–3420 bp; GenBank accession number, XM_032905621.1), *Kcna2* (2309–3063 bp; XM_006233133.3) and *Kcna6* (1368–1868 bp; NM_023954.1) mRNA. Riboprobes were synthesized by *in vitro* transcription using the Bluescript II plasmid vector encoding the above cDNAs, as described previously.^20^ Rats were deeply anaesthetized with a mixture of medetomidine hydrochloride (1.5 mg/kg), midazolam (8 mg/kg) and butorphanol (10 mg/kg) dissolved in saline. They were then transcardially fixed with 4% paraformaldehyde in 0.1 M PB (pH 7.2), followed by decapitation and postfixing for 1 day in the same fixative. After cryoprotection with 30% sucrose in 0.1 M PB, sections (50 μm) were cut on a cryostat (CM1900; Leica Microsystems, Germany). After acetylation and prehybridization incubation, free-floating sections were hybridized with a fluorescein-labelled cRNA probe (diluted with hybridization buffer at 1:1000) at 63.5°C overnight. Following stringent post-hybridization washes, fluorescein was visualized using a peroxidase-conjugated anti-fluorescein antibody (1:1000, 1.5 h; Roche Diagnostics, Tokyo, Japan) and an FITC-TSA Plus amplification kit (PerkinElmer, USA). After extensive washing and blocking with 10% donkey serum, sections were incubated with a mixture of mouse anti-NeuN (MAB377; Merck Millipore, CA, USA) and rabbit anti-tryptophan hydroxylase (TPH)2 (1 µg/ml each) overnight. Finally, sections were incubated with Alexa 647-conjugated anti-mouse IgG (Invitrogen, Carlsbad, CA, USA) and Alexa 568-conjugated anti-rabbit IgG (Invitrogen) for 2 h. Images were taken with a laser scanning microscope (FV1200; Olympus, Tokyo, Japan) equipped with 473, 559 and 635 nm diode laser lines and UPlanSApo (20×/0.75) objective lenses (Olympus). All images represent single optical sections.

### Drugs

DTX-I (#4330-s) and apamin (#4257-v) were obtained from The Peptide Institute (Osaka, Japan). Hepatopodatoxin-2 (#STH-340) was obtained from Alomone Labs (Jerusalem, Israel). Tetraethylammonium (TEA) was obtained from Nacalai Tesque (Kyoto, Japan). Ketamine hydrochloride (#GYA0042) was obtained from Daiichi Sankyo Propharma (Tokyo, Japan).

### Statistical analysis

Five-week-old male Sprague Dawley rats were used in the experiments. We checked the variability resulting from individual differences among the rats using a generalized linear mixed model, and confirmed that the incorporation of individual rat variability as a random effect did not improve the goodness of fit (Supplementary Fig. 1). ‘*n*’ represents the number of DRN neurons and ‘rats’ represents the number of rats. The numbers of DRN neurons recorded in individual rats were 1–5 in non-LH, 1–4 in LH and 1–6 in naïve rats. In pharmacological experiments, data from control and drug-treated DRN neurons were sampled from the same animals. The sample sizes for the experiments were chosen based on previous studies involving similar experiments. The data in the figures are presented as mean ± SEM. The data in the text and tables are presented as mean ± SD. Statistical comparisons between two samples were performed by *t*-test or the Mann–Whitney *U*-test, depending on whether the datasets passed the normality test and equal variance test, unless otherwise stated in the text. Statistical comparisons among three or more groups were assessed by one-way ANOVA. When the difference was significant, data were processed using the Holm–Šidák test as a *post hoc* test. All tests except Fisher’s exact test were two-sided. Data handling and statistical analyses were performed using Excel (Microsoft), Origin 2018 (LightStone), SPSS v27 (IBM), Igor Pro 6.3.7 (WaveMetrics) and SigmaPlot 12.1 (Systat Software). Differences between two samples were considered statistically significant if the *P*-value was lower than 0.05.

### Data availability

Data analysed in this study are available from the corresponding author on reasonable request.

## Results

### Activity-induced 5-HT release is attenuated in LH rats

We exposed rats to inescapable foot shocks using the procedure described in our previous paper.^21^ After the AT session, rats were classified as ‘non-LH’, ‘intermediate’ and ‘LH’ rats (see Materials and methods section). Rats that were placed under the same conditions without foot shocks were classified as ‘naïve’. We used naïve, LH and non-LH rats in the following experiments.

**Figure 1:**
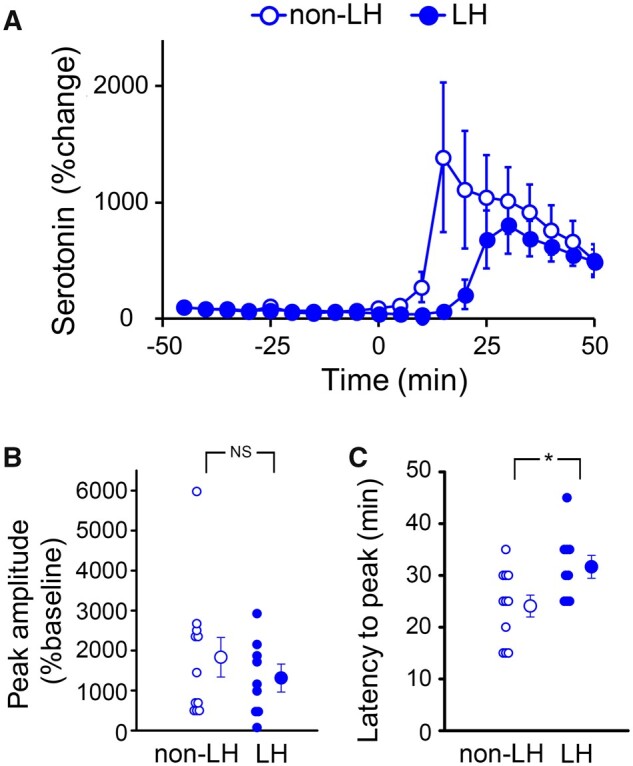
**Chemically stimulated 5-HT release is downregulated in the DRN of LH rats.** (**A**) Line plots of the mean extracellular concentration of 5-HT (as represented by change in percentage from the baseline) in the DRN of non-LH (open circles, rats =11) and LH rats (closed circles, rats = 9), before and after stimulation by the high-K^+^ solution applied at time zero. (**B and C**) Individual data points and the mean peak amplitude (*P* = 0.494, Mann–Whitney *U*-test) (**B**) and latency to peak [*t*(18) = −2.47, *P* = 0.024, *t*-test] (**C**) of the extracellular 5-HT concentration in the DRN of non-LH (open) and LH rats (closed) after chemical stimulation. Data are presented as mean ± SEM. *P<0.05.

First, we examined whether the 5-HT release evoked by the activation of DRN neurons was changed in LH rats. The 5-HT release was induced by the local administration of high-K^+^ solution to the DRN via a dialysis probe. We simultaneously measured the 5-HT level in the DRN with a microdialysis probe inserted with a guide cannula. Administration of the high-K^+^ solution increased the 5-HT level relative to the baseline in the DRN of both non-LH and LH rats (Fig. 1). Analysis of the temporal changes in the extracellular 5-HT level revealed that the peak latency of the 5-HT increase was significantly longer in LH rats (Fig. 1A and C and Supplementary Fig. 2A). While the peak amplitude ratio relative to the baseline did not differ significantly between these groups, it was substantially smaller in LH rats than in non-LH rats (Fig. 1A and B and Supplementary Fig. 2A). These results suggest that the activity-dependent 5-HT release rate is slower in LH rats.

### Subthreshold electrophysiological properties are identical among naïve, non-LH and LH rats

**Figure 2:**
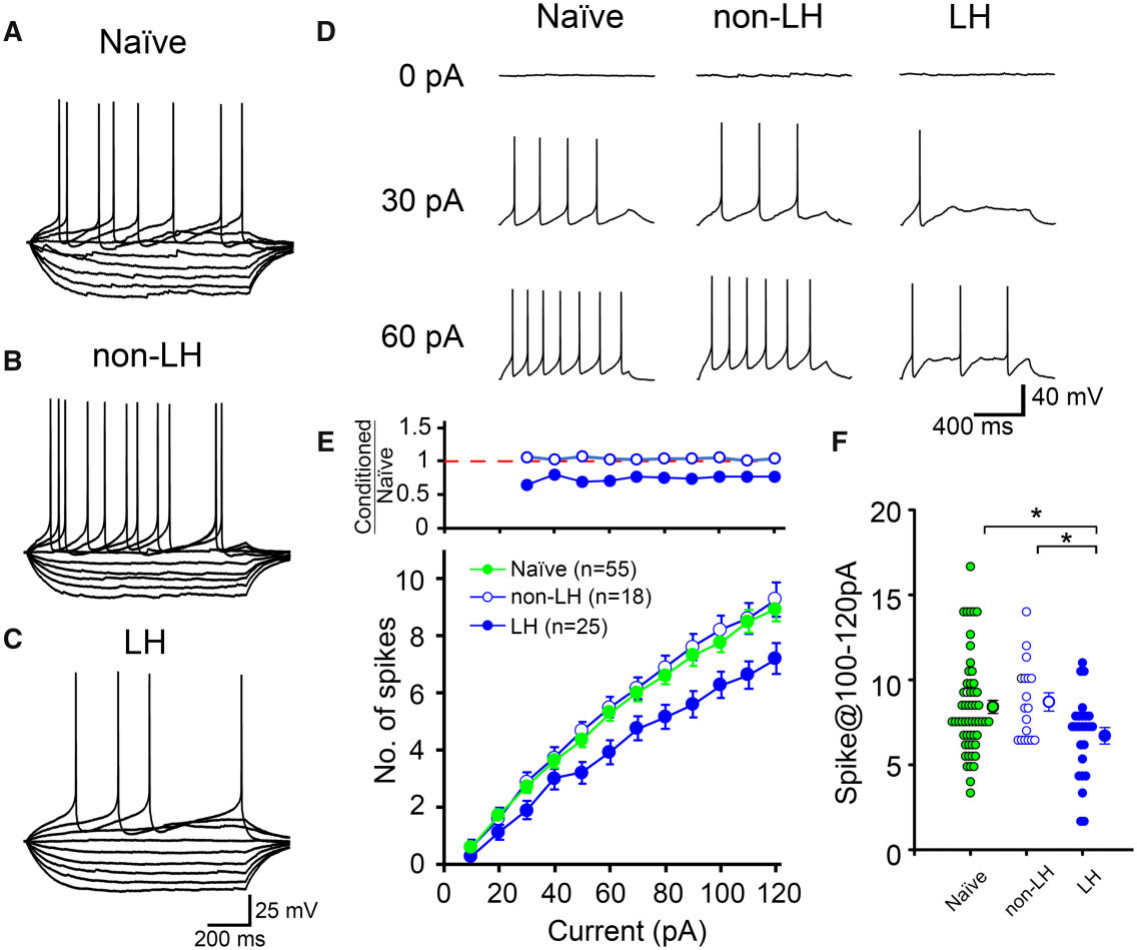
**Spike firing of 5-HT neurons is attenuated in LH rats.** (**A–C**) Representative voltage traces in response to 10 pA steps of hyper- and depolarizing current injections (−50 to +30 pA) in naïve (**A**), non-LH (**B**) and LH (**C**) rats. (**D**) Representative voltage traces in response to depolarizing current injections of 0 pA (upper), 30 pA (middle) and 60 pA (bottom) into naïve (left), non-LH (middle) and LH (right) rats. (**E**) Average spike numbers are plotted against the amplitudes of the injected currents. Green solid, blue open and blue solid symbols represent data of naïve (*n*=55 cells, rats =23), non-LH (*n*=18 cells, rats =7) and LH rats (*n*=25 cells, rats =13), respectively. Recordings of all neurons in non-LH (7 animals) rats and 18 out of 25 neurons in LH (8 out of 13 animals) rats were performed in a manner blinded to the phenotype. (Upper) Ratio of average spike number in conditioned rats (non-LH and LH) relative to that in naïve rats in the lower panel. (**F**) Individual data and average spike numbers upon 100, 110 and 120 pA current injections (spikes@100–120 pA). *P < 0.05, one-way ANOVA, *post hoc*, Holm–Šidák test.

The local injection of high-K^+^ solution to the DRN depolarizes membrane potential and triggers spike firing in somata of 5-HT neurons, which subsequently induces 5-HT release from presynaptic terminals. Therefore, slow 5-HT release may be explained by the attenuation of depolarization-induced spike firing at somata of 5-HT neurons in LH rats compared with the level in non-LH rats. To test this possibility, we examined the electrophysiological properties of 5-HT neurons. Whole-cell recordings were performed from neurons in the ventro-medial part of the DRN above the decussation of the superior cerebellar peduncle. This DRN region is reported to involve 5-HT neurons that mainly project to anterior brain regions, such as orbital cortex, piriform cortex, olfactory-related brain areas and olfactory bulb.^22^^,^^23^ Voltage responses were recorded in the current-clamp mode. After recording the resting membrane potential, the membrane potential was adjusted at −60 mV and voltage responses were examined (Fig. 2A–C). The resting membrane potential, input resistance and time constants of hyperpolarizing potentials were identical among the naïve, non-LH and LH groups (Table 1 and Supplementary Table 1) and to those reported in previous experiments.^17^^,^^19^ These results suggest that the subthreshold electrophysiological properties were identical among the three rat groups.

**Table 1:** Electrophysiological properties of 5-HT neurons in the DRN

	Resting membrane potential (mV)	Input resistance (MΩ)	*τ* of hyperpolarizing potential (ms)	***n***
Naïve	−55.0 ± 6.8	510.4 ± 132.1	44.2 ± 11.3	16
Non-LH	−54.1 ± 6.5	493.5 ± 74.8	46.2 ± 10.0	18
LH	−52.0 ± 6.5	517.2 ± 99.9	43.2 ± 11.8	25
Naïve[DTX(+)]	−52.8 ± 8.1	414.1 ± 107.9	35.7 ± 9.5	7
Non-LH[DTX(+)]	−57.6 ± 3.0	538.7 ± 133.9	30.8 ± 7.8*	7
LH[DTX(+)]	−52.7 ± 6.6	546.7 ± 122.4	42.2 ± 11.3	13

Resting membrane potential was measured at the start of whole-cell recording. Input resistance was calculated from the current–voltage curve obtained by current injections in 10 mV steps from −10 to −80 pA. The time constant (*τ*) of the hyperpolarizing potential was measured from the voltage trace in response to the −100 pA hyperpolarizing current step (1 s). Statistical significance was assessed by one-way ANOVA and *P* < 0.05 was considered significant. When the difference was significant, data were processed using the Holm–Šidák test as a *post hoc* test. Differences between groups were considered significant at *P < 0.05. Statistical significance of data in the presence of DTX-I ([DTX(+)]) (lower lows) was assessed with data of the corresponding control groups, and only the *τ* of hyperpolarizing potential in ‘non-LH+DTX’ was statistically significant. However, we did not address this point further because it was not associated with the induction of behavioural changes.

### The action potential firing is attenuated in 5-HT neurons in LH rats

We next examined spike firing properties in response to depolarizing current injections. Current steps from 10 to 120 pA (10 pA intervals, 1 s duration) were applied to 5-HT neurons. In all rat groups, most 5-HT neurons started to fire upon the injection of 20 pA current, and spike numbers increased with the amplitude of the input current (Fig. 2D and E and Supplementary Fig. 2B). However, we found that the spike firing was significantly decreased in LH rats (Fig. 2D–F and Supplementary Fig. 2B). The ratios of the average spike numbers in the LH rats relative to those in the naïve rats were similar upon most of the tested input currents (Fig. 2E, upper), suggesting that spike firing was suppressed to a similar proportion irrespective of the magnitude of depolarization. Meanwhile, the spike firing in the non-LH rats was not significantly different from that in the naïve rats (Fig. 2D–F and Supplementary Fig. 2B). These data suggest that spike firing is selectively attenuated in LH rats.

### Identification of the K^+^ channels that regulate the action potential firing in 5-HT neurons

We next examined the mechanisms underlying the firing attenuation. In the LH rats, the resting membrane potential and input resistance were identical to those in the naïve and non-LH rats (Table 1 and Supplementary Table 1), suggesting that the ion channels opening at the resting potential were not affected. For example, the spike firing of 5-HT neurons is reportedly suppressed by 5-HT1A activation that hyperpolarizes the membrane potential and decreases input resistance,^24–28^ which is mediated by inwardly rectifying K^+^ channels.^24^^,^^26^^,^^29^ However, the resting membrane potential and input resistance were identical among the three rat groups (Table 1), and there was no correlation between the resting membrane potential and average spike numbers at 100, 110 and 120 pA current injections (Spikes@100–120 pA) (Supplementary Fig. 3). These findings suggest that 5-HT1A-dependent hyperpolarizing shift of the resting membrane potential did not occur in LH rats under our experimental conditions. Furthermore, the kinetics and threshold voltage of the Na^+^ spikes were largely identical among the three groups (Table 2 and Supplementary Table 2), indicating that the firing attenuation is not caused by changes in the voltage-dependent Na^+^ channels. Therefore, we assumed that the firing attenuation was caused by changes in K^+^ conductance activated by membrane potential depolarization. To test this possibility, we initially identified the K^+^ channels that regulate spike firing using naïve rats.

**Table 2:** Kinetics of action potentials in 5-HT neurons in the DRN

Rat groups	Amplitude (mV)	Half width (ms)	Threshold (mV)	AHP amp (mV)	*n*
Naïve	82.5 ± 4.2	1.0 ± 0.1	−35.2 ± 2.8	−26.2 ± 3.8	16
Non-LH	87.9 ± 6.0	1.0 ± 0.2	−33.3 ± 3.8	−29.2 ± 5.1	18
LH	84.1 ± 6.3	1.1 ± 0.2	−34.4 ± 3.9	−27.7 ± 4.2	25

Action potential kinetics was measured using Minianalysis Program (version 6.0.7, Synaptosoft). Amplitude was measured from the threshold to the peak. Threshold was the threshold membrane potential for the action potential generation. Difference of the action potential amplitude between naïve and non-LH was statistically significant (*P*=0.024, one-way ANOVA *post hoc* Holm–Šidák test), but we did not address this point further in this study because it was not associated with the induction of behavioural changes.

AHP = afterhyperpolarization.

**Figure 3:**
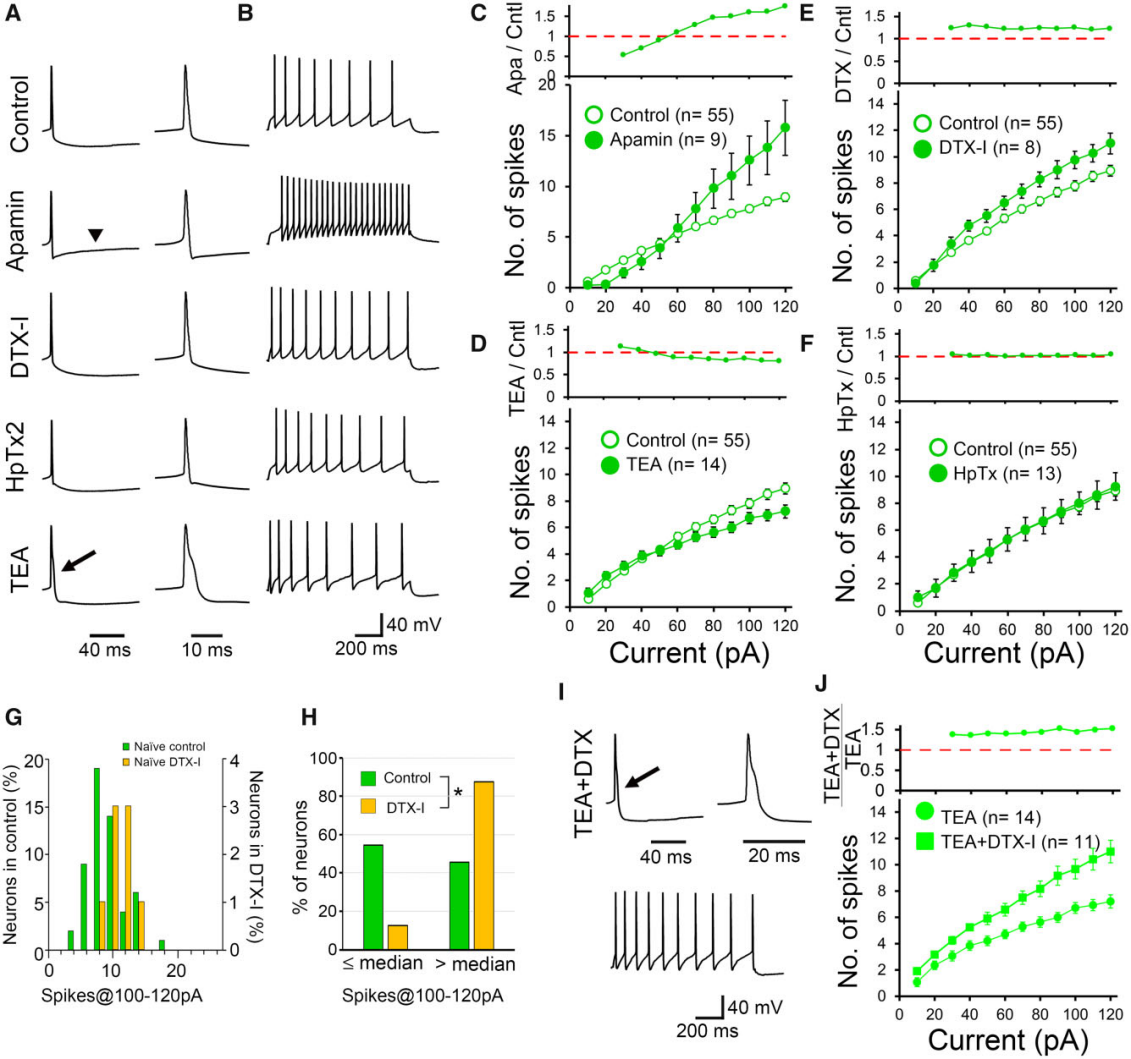
**Contributions of voltage-dependent and Ca^2+^-activated K^+^ channels to the spike firing of DRN 5-HT neurons in naïve rats.** (**A**) (Left) Representative waveforms in normal Ringer solution (control) and in the presence of apamin (100 nM), DTX-I (100 nM), HpTx2 (200 nM) or TEA (500 µM). Afterhyperpolarization was suppressed in the presence of apamin (arrowhead). The width of the action potential was prolonged by 500 µM TEA (arrow). (Right) The time scale of the left traces around the Na^+^-spike was expanded. (**B**) Representative spike trains in response to a current injection of 120 pA. (**C–F**) Average spike numbers in response to depolarizing current injections (10–120 pA with 10 pA intervals, 1 s duration) in normal Ringer solution (open symbols, *n* = 55 cells, rats = 23) and in the presence of apamin (**C**, closed symbols, *n* = 9, rats = 3), TEA (**D**, *n* = 14, rats = 3), DTX-I (**E**, closed symbols, *n* = 8, rats = 2) or HpTx2 (**F**, closed symbols, *n* = 13, rats = 5) are plotted against the amplitudes of the injected currents. (Upper) The ratio of the average spike numbers in the presence of individual blockers relative to those in the normal external solution in individual lower panels. (**G**) Frequency distribution histogram of the average spike numbers upon 100 − 120 pA current injections (spikes@100–120 pA) (*n* = 55, rats = 23 for control and *n* = 8, rats = 2 for DTX-I). (**H**) Frequency distributions of the proportion of 5-HT neurons with spikes@100–120 pA higher than or equal to/lower than the median spikes@100–120 pA (7.67) in normal external solution (control). The proportion of 5-HT neurons with average spike numbers higher than the median was significantly increased in the presence of DTX-I [*P* = 0.029, Fisher’s exact test (one-sided)]. (**I**) Representative waveforms of a single spike and spike trains upon the injection of 120 pA current. DTX-I enhanced the spike firing even in the presence of 500 μM TEA. (**J**) Similar to **E**, but data in the presence of TEA alone (same as data in **D**) or TEA plus DTX-I (*n* = 11, rats = 3).

Small conductance Ca^2+^-activated K^+^ (SK) channels are activated by Ca^2+^ influx caused by membrane potential depolarization, and participate in the generation of afterhyperpolarization.^30^*Kcnn2* and *Kcnn3* are strongly expressed in the DRN.^31^ Bath-applied apamin (100 nM), a specific blocker for SK channels, suppressed the afterhyperpolarization (Fig. 3A, arrowhead and Supplementary Fig. 4A) and enhanced the spike firing elicited by larger current injections (Fig. 3B and C and Supplementary Fig. 2C). These data suggest that afterhyperpolarization is mediated by the SK channel in DRN 5-HT neurons.^32^^,^^33^

The repolarization of the action potential is accelerated by the activation of Kv3, high voltage-activated voltage-dependent K^+^ channels,^34^^,^^35^ and/or the rapid afterhyperpolarization mediated by the big conductance Ca^2+^-activated K^+^ (BK) channels.^35^^,^^36^ We tested the contribution of these channels using a relatively low concentration of TEA (500 μM), which suppresses Kv1.1, Kv3, Kv7 and BK channels.^37^^,^^38^ Bath-applied 500 μM TEA prolonged the decay phase of spikes (Fig. 3A, arrow and Supplementary Fig. 4B). Although the DRN has not been identified to highly express Kv3^39^ or BK^40^ channels, the present results suggest that 500 μM TEA-sensitive channels cause the rapid repolarization of spikes. The ratio of firing with and without TEA was substantially decreased at strong depolarizations (Fig. 3D and Supplementary Fig. 2C), probably because of poor recovery from the Na^+^ channel inactivation.^34^^,^^35^

Kv1 channels are low voltage-activated voltage-dependent K^+^ channels that contribute to suppressing the spike firing of neurons.^30^^,^^41–43^ Kv1.1 and Kv1.2 are weakly expressed in the DRN.^44^^,^^45^ In this study, DTX-I (100 nM), a blocker of Kv1.1, 1.2 and 1.6 channels,^41^^,^^42^ did not affect the resting membrane potential and input resistance (Table 1). However, it slightly enhanced the firing frequency (Fig. 3A, B and E and Supplementary Fig. 2C) to similar proportions upon injection of most of the tested currents (Fig. 3E, upper). The distribution of spikes@100–120 pA in the DTX-I solution was narrower than that in the control solution, and largely overlapped at the higher firing frequency range in the control solution (Fig. 3G). This suggests that DTX-I preferentially enhances the spike firing of neurons with a lower frequency, but this effect is less significant in those firing at a higher frequency. To investigate this further, neurons were classified into two groups, those with more than or equal to/less than the median number of spikes@100–120 pA of naïve rats in the control solution. As expected, DTX-I significantly decreased the percentage of neurons firing at a lower frequency (Fig. 3H). These findings suggest that DTX-I-sensitive Kv1 channels are expressed only in a subset of 5-HT neurons in the DRN in naïve rats. We also examined the effect of heteropodatoxin-2 (HpTx2) (200 nM), a Kv4 blocker, on spike firing. Although Kv4.3 is expressed in the DRN,^46^ HpTx2 had no effect on the spike firing (Fig. 3A, B and F and Supplementary Fig. 2C).

Taking these findings together, we concluded that the spike firing of 5-HT neurons is regulated by multiple K^+^ channels: 500 μM TEA-sensitive K^+^ channels cause rapid repolarization of the action potential, and the SK channels produce afterhyperpolarization. DTX-I-sensitive Kv1 channels suppress the spike firing during the injection of depolarizing current.

### Firing attenuation is caused by enhancement of DTX-I-sensitive Kv1 channels

Attenuation of the spike firing in LH rats occurred upon injection of most of the tested currents at similar proportions, even the smaller ones (Fig. 2E). This suggests that the activation voltage of candidate ion channels is near the resting membrane potential (low voltage activation). Among the candidate K^+^ channels, Kv1 is likely the most dominant because of its low voltage-activated property.^41–43^ This low voltage activation was also confirmed by the data showing that DTX-I equally enhanced the spike firing irrespective of the magnitude of depolarization (Fig. 3E, upper). In contrast, this low voltage-activated property excluded Kv3 from the list of candidates. Additionally, Kv3 or BK modulation changed the spike duration (Fig. 3A and Supplementary Fig. 4B), but this was not observed in the LH rats (the half width in Table 2). Similarly, SK channels were also excluded. Change in SK channels should accompany an increase or decrease of the afterhyperpolarization of spikes (Fig. 3A and Supplementary Fig. 4A), but the afterhyperpolarizations did not differ among the three rat groups (Table 2). Taking these findings together, we thought that the firing attenuation was caused by DTX-I-sensitive Kv1 modulation.

**Figure 4:**
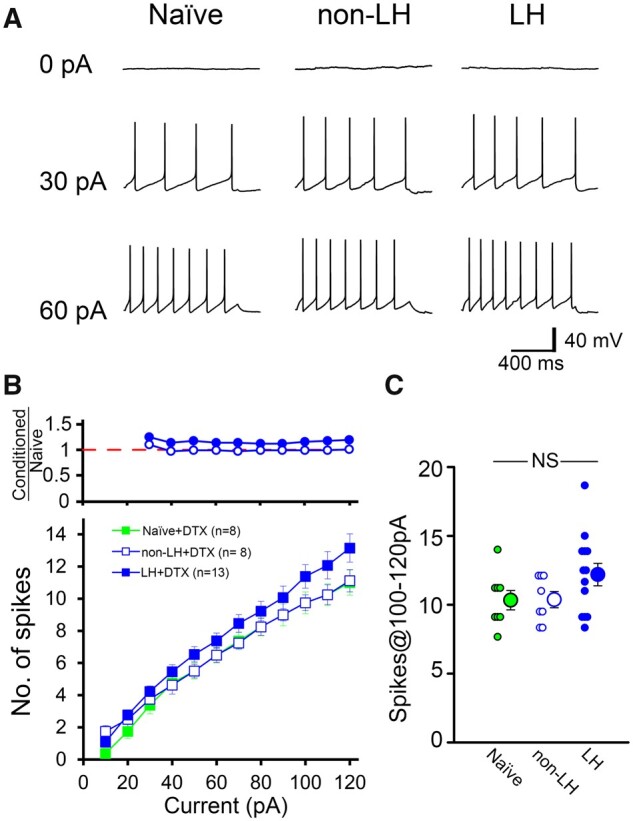
**Firing attenuation in LH rats is reversed in the presence of DTX-I.** (**A**) Representative voltage traces in response to depolarizing current injections of 0 pA (upper), 30 pA (middle) and 60 pA (bottom) in naïve (left), non-LH (middle) and LH (right) rats in the presence of DTX-I (100 nM). (**B**) The average spike numbers in the presence of DTX-I are plotted against the amplitudes of the depolarizing currents. Blue open and blue solid symbols represent data of non-LH (*n*=8 cells, rats = 2) and LH (*n*=13 cells, rats = 4) rats, respectively. The naïve rats’ data (green closed squares) are the same as in Fig. 3E (green closed circles). (Upper) The ratio of the average spike numbers of conditioned rats (non-LH and LH) relative to that of naïve rats in the lower panel. (**C**) Individual data and the average spikes@100–120 pA. There were no statistically significant differences [*F*(2, 26)=2.118, *P*=0.141, one-way ANOVA].

We examined the effects of DTX-I on the spike firing in non-LH and LH rats (Fig. 4 and Supplementary Fig. 2D). Similar to naïve rats, bath-applied DTX-I facilitated the generation of spikes in both non-LH and LH rats (Fig. 4 and Supplementary Fig. 2D). Notably, the enhancement was prominent in LH rats and the spike firing frequency increased above the levels of naïve and non-LH rats in control external solution: spikes@100–120 pA of naïve[DTX(−)]: 8.4 ± 2.9 (*n* = 55, rats = 23); non-LH[DTX(−)]: 8.7 ± 2.3 {*n* = 18, rats = 7, versus naïve[DTX(–)]: *P* = 0.699}; and LH[DTX(+)]: 12.2 ± 2.9 {*n* = 13, rats = 4, versus naïve[DTX(−)]: *P* < 0.001, versus non-LH[DTX(−)]: *P =* 0.002, *df = 2, F = 10.083*, *P < 0.001*; one-way ANOVA *post hoc* Holm–Šidák test}. The results showed that the difference in spike firing among rat groups disappeared in the presence of DTX-I (Fig. 4B and C and Supplementary Fig. 2D). These findings suggest that DTX-I-sensitive Kv1 activity is significantly enhanced in 5-HT neurons in LH rats. In turn, the enhanced Kv1 likely attenuates the spike firing.

**Figure 5:**
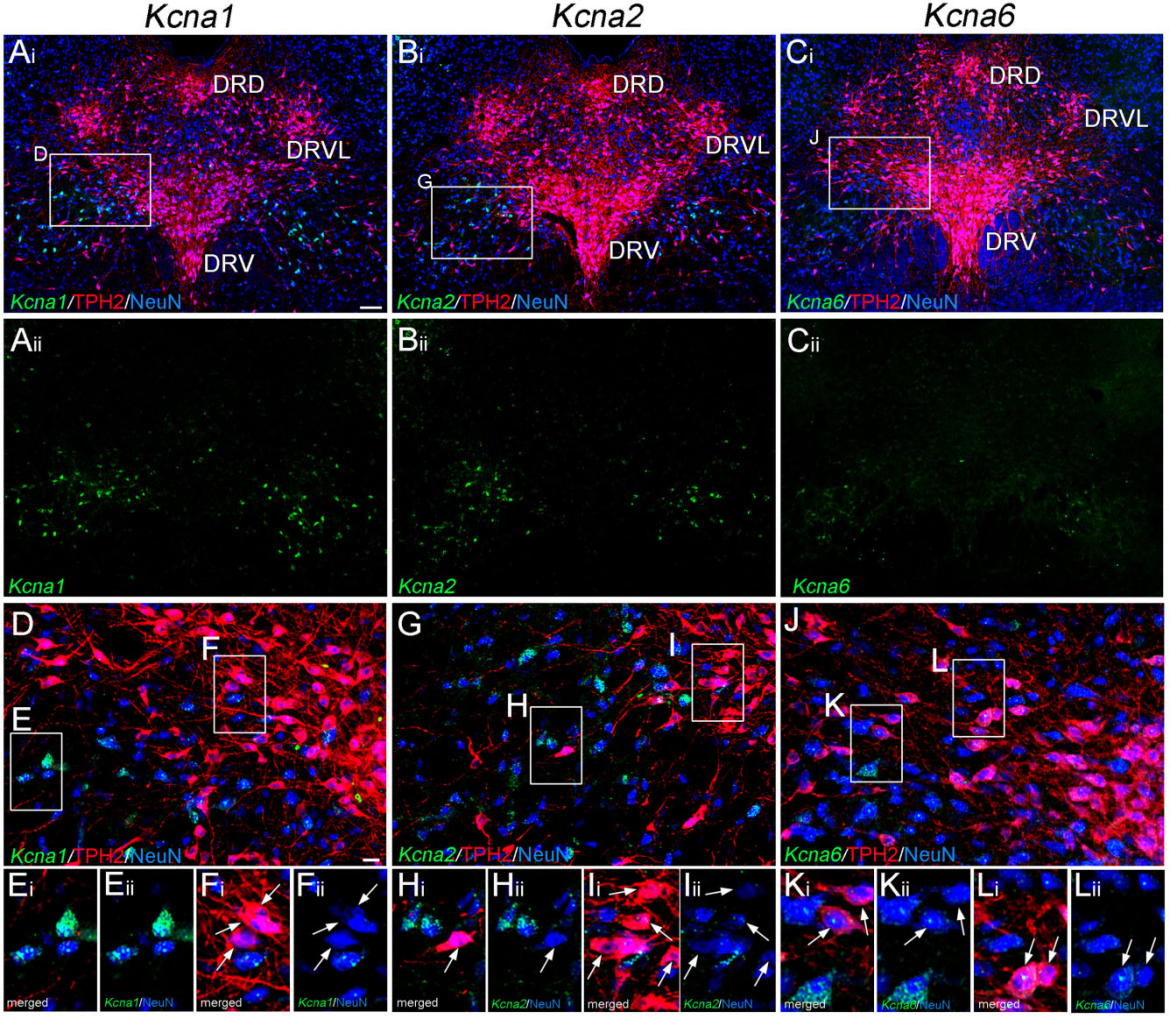
**DTX-I-sensitive Kv1 channels are expressed in DRN neurons.** (**A–C**) Fluorescent *in situ* hybridization combined with immunolabeling of TPH2 (red) and NeuN (blue) showing expression of *Kcna1* (Kv1.1, **A**), *Kcna2* (Kv1.2, **B**) and *Kcna6* (Kv1.6, **C**) mRNA (green) in the DRN. A(i)–C(i), all merged images; A(ii)–C(ii), Kv1 signals. (**D, G and J**) Magnified images of the square areas in A(i), B(i) and C(i). (**E–L**) Magnified images of the square areas in **D**, **G** and **J**. E(i)–L(i), all merged images; E(ii)–L(ii), merged images of Kv1 and NeuN signals. Arrows indicate TPH2-positive neurons. Scale bars, A(i), 100 μm; **D**, 20 μm.

We next examined the subtypes of DTX-I-sensitive Kv1 channels (Kv1.1, Kv1.2 and Kv1.6) expressed in 5-HT neurons in the naïve rat DRN using *in situ* hybridization (Fig. 5). As expected from the electrophysiological data (Fig. 3G-H), the expression of these Kv1 channels in DRN nuclei was weak and sparse in naïve rats (Fig. 5). The signals of Kv1.1, Kv1.2 and Kv1.6 mRNA were very weak in TPH2-positive 5-HT neurons (Fig. 5D–L). These results suggest that Kv1.1, Kv1.2 and Kv1.6 are expressed in the DRN, although the overall expression levels are weak in naïve 5-HT neurons.^44^^,^^45^

We investigated changes in the Kv1 expression pattern in the DRN using immunohistochemical staining. We examined Kv1.1 and Kv1.2 because reliable antibodies were available for them. Both Kv1.1 and Kv1.2 antibodies presented staining in filamentous and diffuse patterns in the DRN (Supplementary Fig. 6A and C). This filamentous staining likely represented the distribution at axon initial segments, but did not colocalize with TPH-positive 5-HT neurons (Supplementary Fig. 6A). The weak and diffuse distribution pattern of Kv1.2 was observed in the entire DRN in non-LH rats (Supplementary Fig. 6A and B). In contrast, the Kv1.2 signal enhanced and exhibited a clear punctate distribution in the entire DRN in LH rats (Supplementary Fig. 6A and B). Some Kv1.2 signals closely apposed the TPH signals (Supplementary Fig. 6B). Enhancement of the Kv1.2 expression also occurred in organs originating from TPH-negative cells (Supplementary Fig. 6A, B and D), suggesting that the Kv1.2 expression was enhanced in the entire DRN by the LH induction. In contrast, the distributions of Kv1.1 were similar in non-LH and LH rats (Supplementary Fig. 6C and E). These morphological findings suggest that the LH induction changes the pattern of Kv1.2 expression in the DRN.

### Ketamine enhances spike firing of 5-HT neurons by suppressing DTX-I-sensitive Kv1 channels

**Figure 6:**
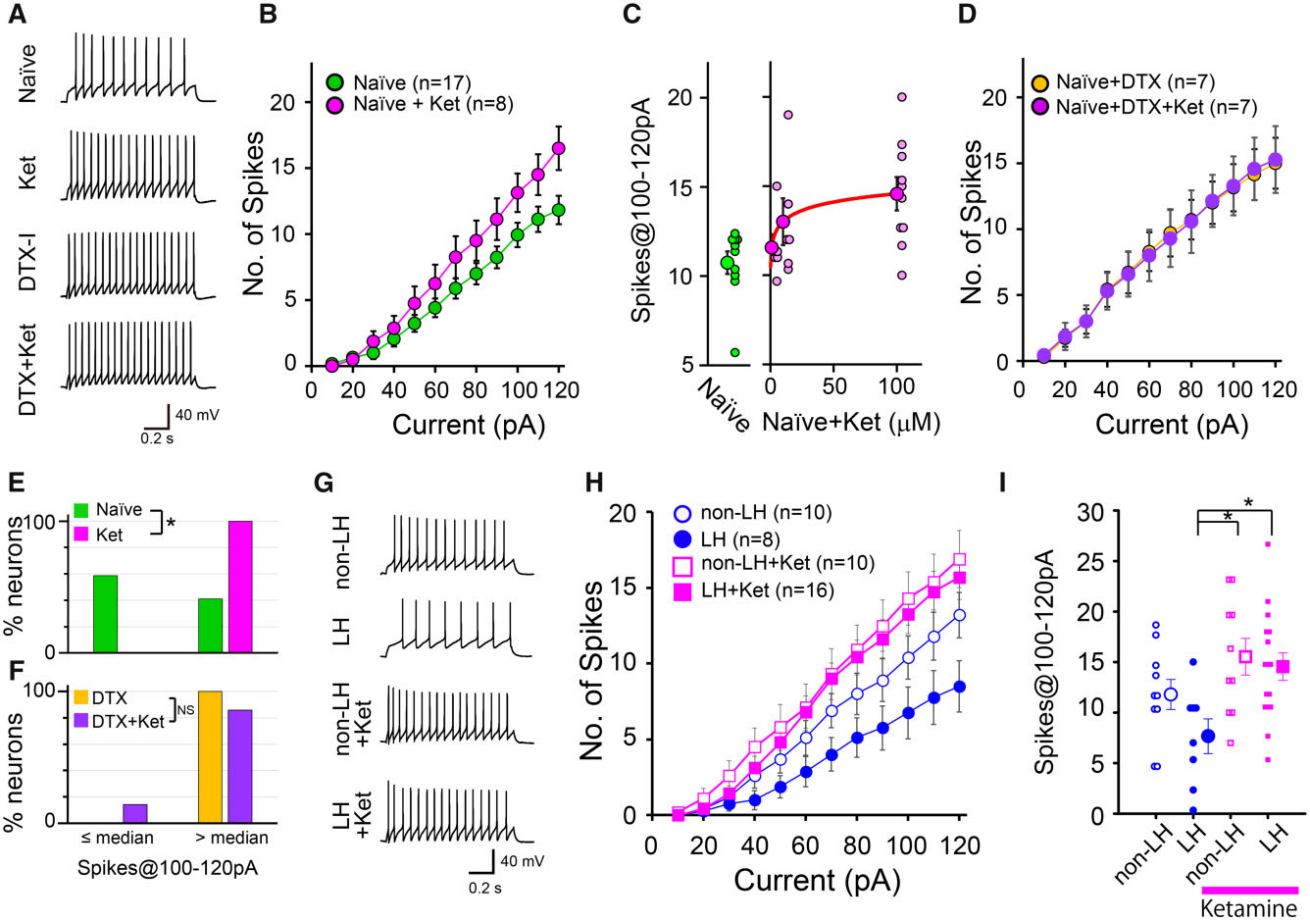
**Ketamine (Ket) facilitates spike firing of 5-HT neurons via suppression of DTX-I-sensitive Kv1 channels.** **(A)** Representative waveforms in response to current injections of 120 pA in normal Ringer solution (naïve) and in the presence of Ket (100 μM), DTX-I (100 nM) or DTX-I and Ket (DTX+Ket) in naïve rats. (**B**) The average spike numbers in response to depolarizing current injections (10–120 pA with 10 pA intervals, 1 s duration) in normal Ringer solution (green symbols, *n*=17 cells, rats =9) and in the presence of Ket (pink symbols, *n*=8, rats =3) in naïve rats. (**C**) Dose–response plots of the spikes@100–120 pA in control solution (green symbols) and in the presence of 1, 10 and 100 μM Ket (pink symbols) in naïve rats (*n*=10, rats = 6 for control and 100 μM Ket; *n*=6, rats = 4 for 1 and 10 μM Ket). Data were fitted by the Hill equation (red line). (**D**) Similar to **B**, but average spike numbers in the presence of DTX-I (orange symbols, *n*=7, rats =4) or DTX-I and Ket (purple symbols, *n*=7, rats =4). (**E**) Frequency distributions of the proportion of 5-HT neurons with spikes@100–120 pA higher than or equal to/lower than the median average spikes@100–120 pA (11) of the normal external solution. The proportion of 5-HT neurons with spikes@100–120 pA larger than the median was significantly increased in the presence of Ket [*P*=0.006, Fisher’s exact test (one-sided)] in naïve rats. (**F**) Similar to **E**, but comparing between DTX-I and the co-application of DTX-I and Ket. These distributions were not significantly different (*P*=0.5). (**G**) Representative waveforms in response to injection of a current of 120 pA in non-LH and LH rats in the presence or absence of Ket. (**H**) Similar to **B**, but average spike numbers in non-LH and LH rats in the presence (pink square symbols; non-LH, *n* = 10, rats = 5; LH, *n* = 16, rats = 5) or absence (blue circle symbols; non-LH, *n*=10, rats =5; LH, *n*=8, rats =5) of Ket. (**I**) Individual data and average spikes@100–120 pA. *P <0.05, one-way ANOVA, *post hoc*, Holm–Šidák test.

This study indicates that DTX-I-sensitive Kv1 suppresses the spike firing of 5-HT neurons. These channels may be a target for drug development to control the spike firing of 5-HT neurons and the resulting 5-HT level in the brain. Previous reports demonstrated that the acute administration of ketamine, which shows rapid antidepressant effects,^47^^,^^48^ enhances the 5-HT level in the prefrontal cortex^49–54^ and the hippocampus.^55^ Because ketamine is reported to suppress Kv channels,^56^^,^^57^ we assumed that it enhances the spike firing of 5-HT neurons by blocking DTX-I-sensitive Kv1 channels. Bath-applied ketamine hydrochloride enhanced the spike firing in naïve rats (Fig. 6A and B; Supplementary Fig. 5A) in a dose–dependent manner (EC_50_ = 22 μM) (Fig. 6C). Similar to DTX-I, ketamine reduced the incidence of neurons firing at a lower frequency (Fig. 6E). The solution of ketamine at 100 μM contained 0.12 μM benzethonium chloride, but it did not affect the spike firing [spikes@100–120 pA in control (11.3 ± 1.9, *n* = 6, rats = 2) and benzethonium (8.5 ± 4.0, *n* = 6, rats = 2), *P =* 0.310, Mann–Whitney test]. Notably, DTX-I pretreatment completely occluded the enhancement of spike firing by the subsequent application of ketamine at 100 μM (Fig. 6A, D and F; Supplementary Fig. 5B), suggesting that ketamine enhances spike firing by suppressing DTX-I-sensitive Kv1 channels. We finally examined whether ketamine reversed the firing attenuation induced by LH. Bath-applied 100 μM ketamine strongly enhanced the spike firing of 5-HT neurons in LH rats (Fig. 6G–I and Supplementary Fig. 5C). These findings raise the possibility that ketamine-dependent 5-HT release is at least partly mediated by the suppression of DTX-I-sensitive Kv1 channels.

## Discussion

### Spike firing of 5-HT neurons in the DRN is attenuated in LH rats

In this study, we found that the somatic firing activity of 5-HT neurons was attenuated in LH rats (Fig. 2). The firing frequency of neurons in naïve rats was identical to that in non-LH rats (Fig. 2D–F), which suggests that the spike firing attenuation is closely associated with the induction of LH. Firing attenuation of 5-HT neurons in the DRN has also been reported in other model animals that show depression- and anxiety-like behaviours, such as social defeat^58^ and chronic social isolation.^59^ The firing attenuation of 5-HT neurons may be a common phenotype associated with depressive behaviours.

Somatic spike firing attenuation reduces the incidence of presynaptic terminal activation, and likely results in a slower activity-dependent 5-HT release rate (Fig. 1). However, several studies have demonstrated that 5-HT neurons are sensitized after ISs,^9–11^ which persists for at least a day. This sensitization is thought to be caused by reduction and/or desensitization of 5-HT1A receptors on DRN neurons.^60^^,^^61^ These findings appear to contradict our results, but our measurement of 5-HT was performed 3–6 days after the IS session. The release of 5-HT may initially increase and then decrease a few days after the inescapable stress. Alternately, the slow 5-HT release at 3–6 days after the IS session may partly reflect circuit modulations relating to the contextual fear conditioning. Behavioural changes are reported to persist longer (∼1 week) because of fear conditioning with contextual factors, if IS and AT sessions are performed in similar cages.^2^ In this study, the IS and AT sessions were performed in similar cages with a grid floor, and depressive behaviours induced by our protocol persisted for at least 5 days (Supplementary Fig. 7), which is longer than the LH in previous studies (48–72 h).^2^

This study suggests that the intrinsic firing activity of 5-HT neurons is attenuated in LH rats in a Kv1-dependent manner. Meanwhile, it was previously reported that the firing changes are also caused by the activation of inhibitory interneurons. The activity of GABAergic neurons in the DRN is enhanced by social defeat stress, which subsequently attenuates the spike firing of 5-HT neurons in susceptible mice.^58^ Furthermore, activities of higher brain regions that negatively regulate DRN activity, such as the prefrontal cortex and habenula,^62–64^ are also enhanced by uncontrollable stress.^65–67^ Enhanced activity of these higher brain areas could also suppress the firing activity of 5-HT neurons. Spike firing activity of 5-HT neurons may be cooperatively regulated by these extrinsic and intrinsic factors.

### Spike firing attenuation is mediated by enhanced activity of the DTX-I-sensitive Kv1 channels

Our analysis suggests that DTX-I-sensitive Kv1 (Kv1.1, Kv1.2 and/or Kv1.6) upregulation was a major cause of the somatic spike attenuation in LH rats (Fig. 4). The spontaneous firing may also be suppressed in LH rats because the resting membrane potential of 5-HT neurons [−65.5 to −36.5 mV (Table 1)] overlaps with the activation voltage of Kv1 channels.^41–43^

DTX-I-sensitive Kv1 channels controlling spike firing were insensitive to 500 μM TEA in naïve rats (Fig. 3I and J). This suggests that Kv1.2 and/or Kv1.6 may mediate firing attenuation because 500 μM TEA suppresses Kv1.1 (IC_50_ = 300 μM), but not Kv1.2 (560 mM) and Kv1.6 (1.7–7 mM).^30^^,^^37^ Furthermore, Kv1.2, but not Kv1.1, became to show clear clusters in LH rats (Supplementary Fig. 6), which supports this possibility. While immunohistochemical changes in Kv1.6 were not examined in this study, it remains a possibility that Kv1.6 also shows similar distribution changes to Kv1.2 in LH rats.

Previous reports suggested that several K^+^ channels participate in controlling animal mood by modulating the spike firing of DRN neurons. Mice with a defect in TREK-1 (*Kcnk2*), classified as a two-pore domain-type K^+^ channel, show increased spontaneous firing of DRN 5-HT neurons and antidepressant behaviour.^68^ In a mouse model of chronic social isolation, firing of DRN 5-HT neurons was attenuated because of the upregulation of SK3 channels.^59^ The firing of 5-HT neurons is finely regulated by several K^+^ channels, and their disorder may participate in the pathogenesis of depressive behaviours.

### Ketamine enhances the spike firing of 5-HT neurons

Ketamine and/or its metabolites have rapid-onset antidepressant effects on model animals of LH and social defeat stress.^48^^,^^69–71^ This study suggests that ketamine blocks the DTX-I-sensitive Kv1 channel. However, it is likely difficult to explain the chronic antidepressant effect by this blocking effect because this effect of ketamine is attenuated with the progression of ketamine decomposition. Meanwhile, several previous reports demonstrated that intraperitoneal (i.p.) or subcutaneous (s.c.) administration of 5–30 mg/kg ketamine causes an acute increase of 5-HT in the brain.^49–55^ It is proposed that the acute ketamine-dependent release of 5-HT is caused by excitatory and/or cholinergic inputs^50^^,^^52^^,^^53^ to the DRN or the decrease in serotonin transporter activity.^72^ In addition to these factors, this study suggests the contribution of spike firing enhancement by the suppression of DTX-I-sensitive Kv1 channels (Fig. 6). According to previous studies using mice, the plasma concentrations in response to i.p. administration of 10 or 30 mg/kg (R)- ketamine within 30 min was 210–333 ng/ml (0.9–1.4 μM) or 757–958 ng/ml (3.2–4.0 μM), respectively.^73–75^ Its i.v. administration to rats tended to result in a higher plasma concentration [3430 ng/ml (14.4 μM) 10 min after 20 mg/kg administration].^75^^,^^76^ Because the content of ketamine in the brain is reported to be higher (2–6 times) than that in the plasma,^73^^,^^76–78^ the actual brain concentration may be higher than these estimations. In this study, the dose–response plots suggested that 10 μM ketamine partially facilitated the spike firing (Fig. 6C). Taking these findings together, it appears that the spike firing can be enhanced by the administration of 10–30 mg/kg ketamine. This study suggests that the DTX-I-sensitive Kv1 channels may be potential targets to control the firing activity of 5-HT neurons.

## Supplementary material

Supplementary material is available at *Brain Communications* online.

## Supplementary Material

fcab285_Supplementary_Data

## Abbreviations

AT =: avoidance test
BK channel =: big conductance Ca^2+^-activated K^+^ channel
DRN =: dorsal raphe nucleus
DTX-I =: dendrotoxin-I
HpTx2 =: heteropodatoxin-2
IS =: inescapable shock
LH =: learned helplessness
SK channel =: small conductance Ca^2+^-activated K^+^ channel
TEA =: tetraethylammonium
TPH2 =: tryptophan hydroxylase 2
5-HT =: 5-hydroxytryptamine (serotonin)
