# Supplementary material for: Spike firing attenuation of serotonin neurons in learned helplessness rats is reversed by ketamine
Source: Brain Commun. 2021 Dec 1;3(4):fcab285. doi: 10.1093/braincomms/fcab285 (PMC8688795; doi:10.1093/braincomms/fcab285)
Supplement: fcab285_Supplementary_Data [file fcab285_supplementary_data.pdf]

Supplementary Methods

Immunostaining

Rats were deeply anesthetized with a mixture of medetomidine hydrochloride (1.5 mg/kg, Domitor; Nippon Zenyaku Kogyo Co., Ltd., Fukushima, Japan), midazolam (8 mg/kg, Sando; Sando Co., Ltd., Aichi, Japan) and butorphanol (10 mg/kg, Vetorphale; Meiji Seika Pharma Co., Ltd., Tokyo, Japan) dissolved in saline. After the transcardial perfusion of 0.9% saline, rats were transcardially fixed with 3% glyoxal (#128465 Glyoxal Solution, Sigma- Aldrich Co. LLC, St. Louis, U.S.A.) (pH 4)[1]. After overnight post-fixation in the same fixative, brains were incubated with 30% sucrose in 0.1 M PB. Coronal sections (50 μm thick) were cut with a microslicer (ZERO-1; Dosaka, Kyoto, Japan). The primary antibodies were anti-Kv1.1 (1:200, RRID: AB_2571787; Nittobo Medical, Tokyo, Japan), anti-Kv1.2 (1:200, RRID: AB_2571789; Nittobo Medical) and anti-tryptophan hydroxylase (Tph; 1:500, RRID: AB_90754; Merck Millipore, CA, USA). Sections were incubated with 10% normal donkey serum for 30 min, followed by a mixture of primary antibodies overnight at 4°C and a mixture of Alexa Fluor 488 and Alexa Fluor 568 (Invitrogen, Carlsbad, CA, USA) species-specific secondary antibodies for 2 h at a dilution of 1:500 at room temperature. Images were taken with a laser scanning microscope (LSM700, Zeiss, Oberkochen, Germany) using a PlanApoN (60×/1.40 oil immersion) objective lens (Zeiss). Images were digitized at 8-bit resolution. Individual mean fluorescent intensities per pixel were calculated from six consecutive optical dorsal raphe nuclei (DRN) images (320 × 320 μm) taken along the z axis at an interval of 1 μm.

Supplementary Reference

1. Richter KN, Revelo NH, Seitz KJ*, et al*. Glyoxal as an alternative fixative to formaldehyde in immunostaining and super-resolution microscopy. *EMBO J.* Jan 4 2018;37(1):139-159. doi:10.15252/embj.201695709

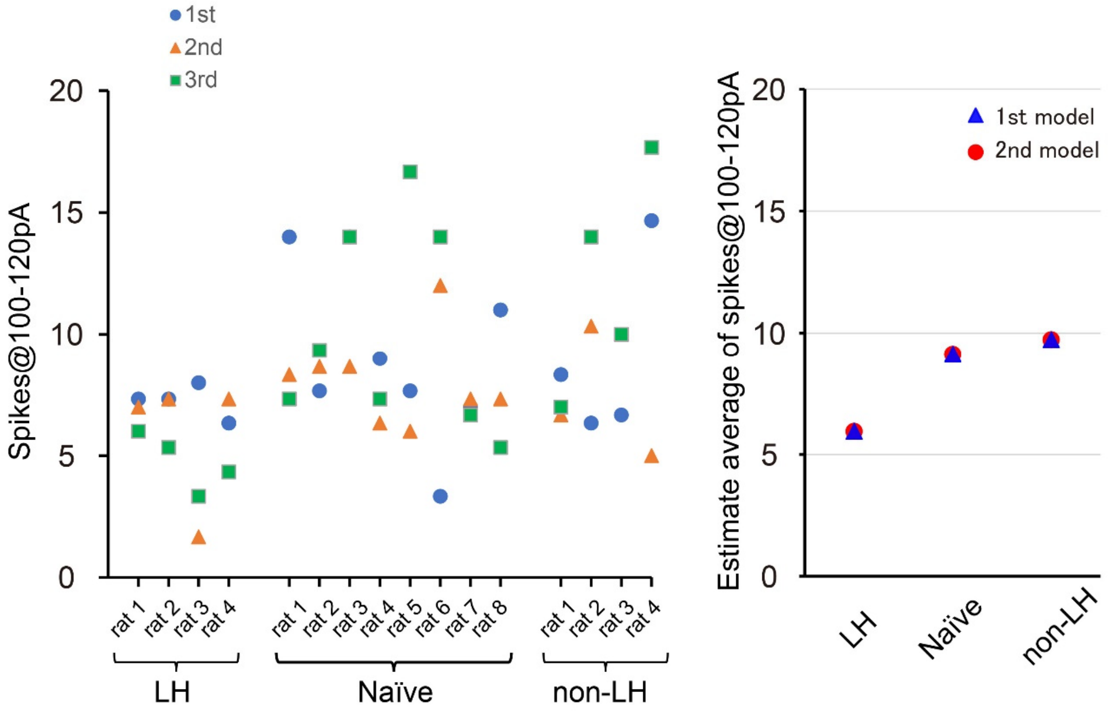

Supplementary Figure 1. Difference in averaged spike numbers among rat groups

Biological variability by the repeated sampling of 5-HT neurons from rats was assessed by a generalized linear mixed model (GLMM) using SPSS. (left) Averaged spike numbers at 100–120 pA (spikes@100–120 pA) of first, second and third DRN neurons sampled in individual rats are plotted for naïve (rats=8), non-LH (rats=4) and LH (rats=4) rat groups. For this analysis, only rats in which three or more DRN neurons were sampled were used from rats analyzed in Fig. 2. The rat group (naïve, non-LH and LH) and the sampled order (first, second and third) were treated as fixed effects, and individual rats were treated as a random effect in the first model. The averages of spikes@100–120 pA in individual rat groups (blue triangles in the right graph) differed significantly ($F_{2,13}$=5.979, p=0.014). In contrast, the random effect due to individual rat variation was not statistically significant (z=1.454, p=0.146, Wald test). Another fixed effect (sampled order, $F_{2,26}$=0.988, p=0.386) and the interaction of rat group and sampled order (group×sampled order, $F_{4,26}$=0.922, p=0.466) were also not significant.

We also compared this first model with the second model that was fitted using only fixed effects. Average values estimated by the first and second models were same (the right graph). The logarithmic likelihood of the first model was −107.95, as was that of the second model. Conditional AIC (cAIC) of the first and second models were 222.594 and 220.242, respectively. The same likelihood and larger cAIC of the first model suggest that inclusion of the random effect did not improve the goodness of fit.

Taking these findings together, repeated sampling from individual rats does not significantly influence the data variability in this experiment; namely, individual rat variability is negligible relative to individual variability among neurons.

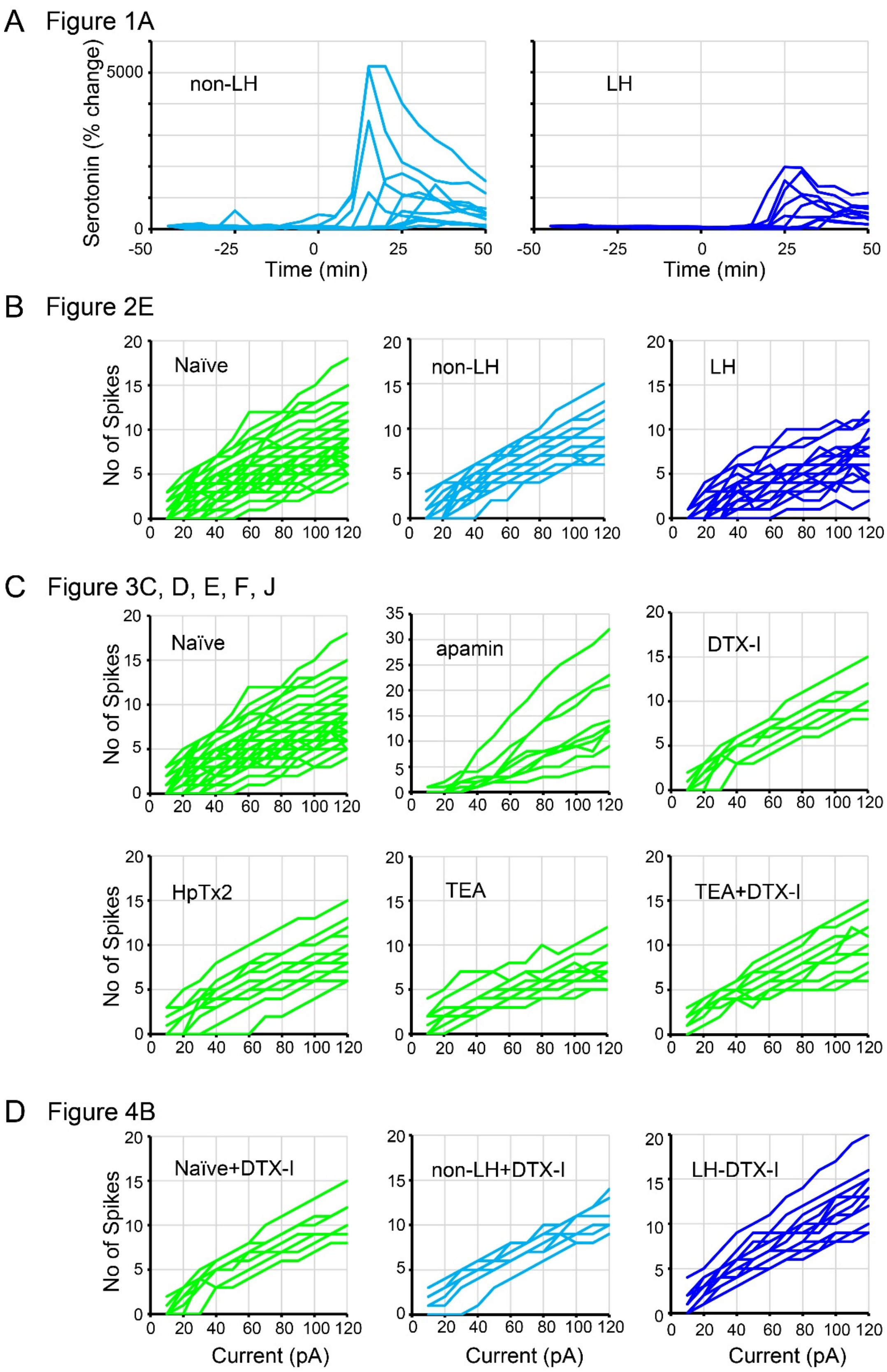

Supplementary Figure 2. Individual data in Figure 1A (A), Figure 2E (B), Figure 3C–J (C) and Figure 4B (D)

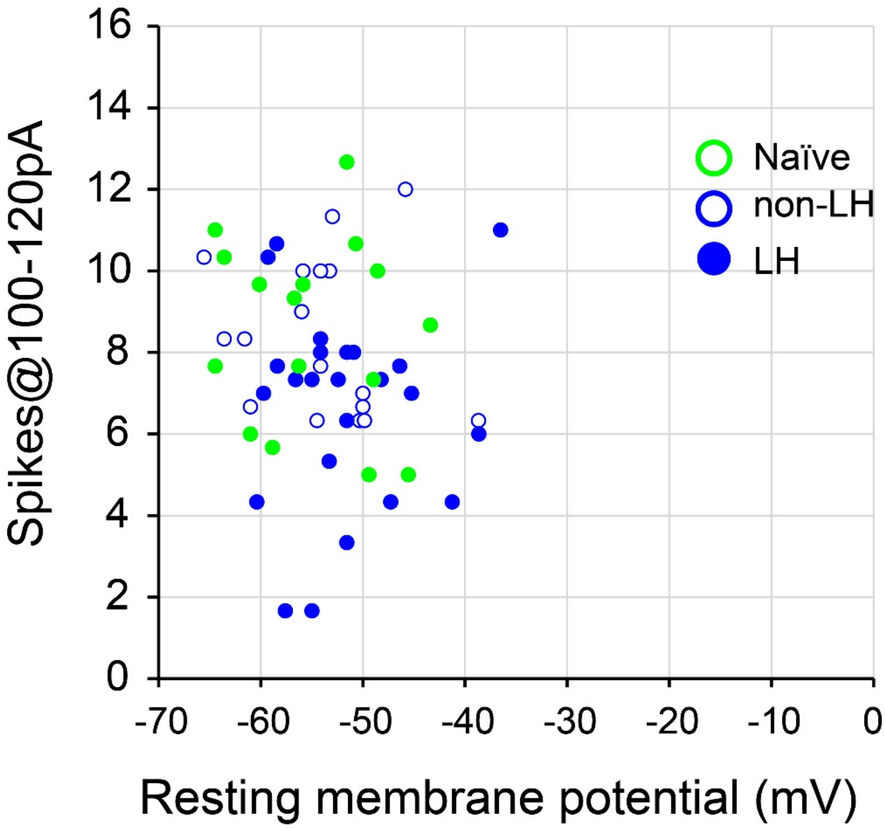

Supplementary Figure 3. Relationship between the resting membrane potential and average spike numbers at 100–120 pA (spikes@100–120 pA)

There was no clear correlation between the resting membrane potential and spikes@100–120 pA [non-LH (n=18, r=–0.265, p=0.298), LH (n=25, r=–0.0324, p=0.876) and naïve (n=16, r=–0.216, p=0.416; Spearman's rank order test].

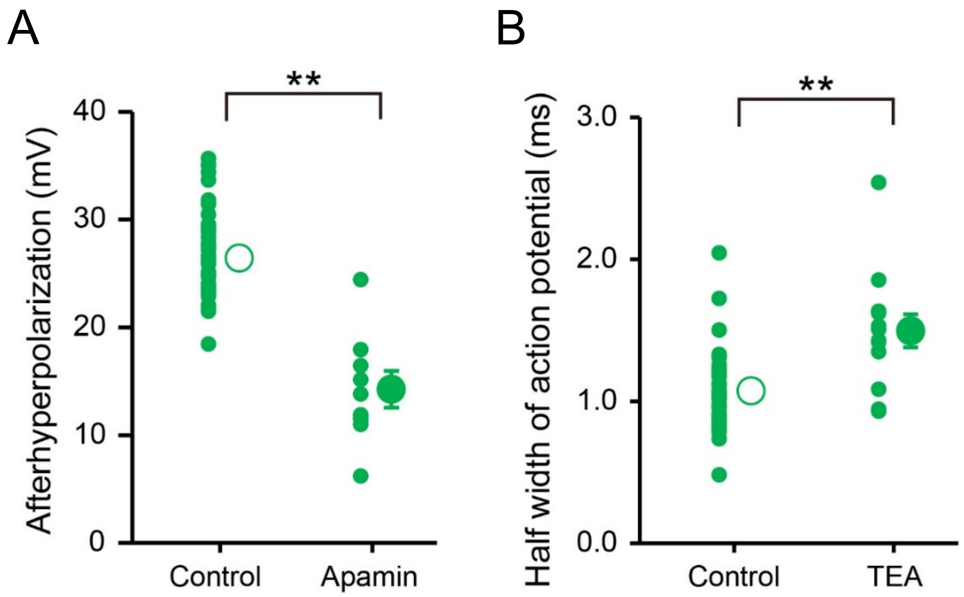

Supplementary Figure 4. Pharmacological effects of apamin and TEA on action potential waveforms

(**A**) The pharmacological effect of apamin on the amplitude of the afterhyperpolarization in naïve rats. Amplitudes of afterhyperpolarization were measured from the action potential threshold to the peak. Afterhyperpolarization was significantly suppressed by apamin (Control, n=37, Apamin, n=9, $p < 0.001$; *t*-test). (**B**) The pharmacological effect of TEA on the half width of the action potential in naïve rats. For this analysis, the half-width values were measured at half amplitude of action potentials measured from the threshold to the peak. These values were slightly shorter than the half-width values measured at half amplitude of action potentials measured from the baseline membrane potential (~–60 mV) to the peak, which was used for identifying 5-HT neurons (Material and Methods, Table 2). The duration was significantly prolonged by TEA (Control, n=37, TEA, n=13, $p<0.001$; Mann–Whitney U-test).

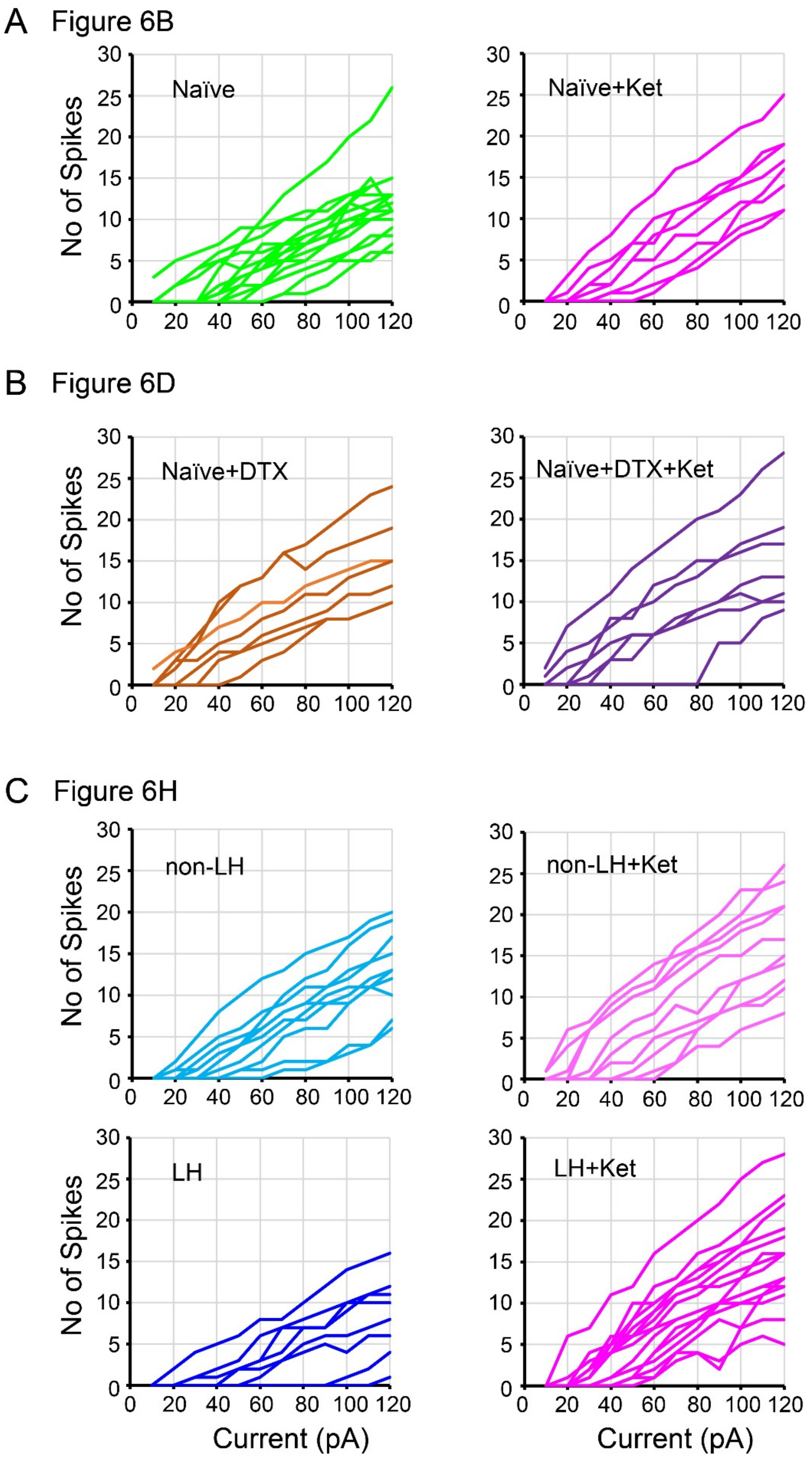

Supplementary Figure 5. Individual data in Figure 6B (A), Figure 6D (B) and Figure 6H (C)

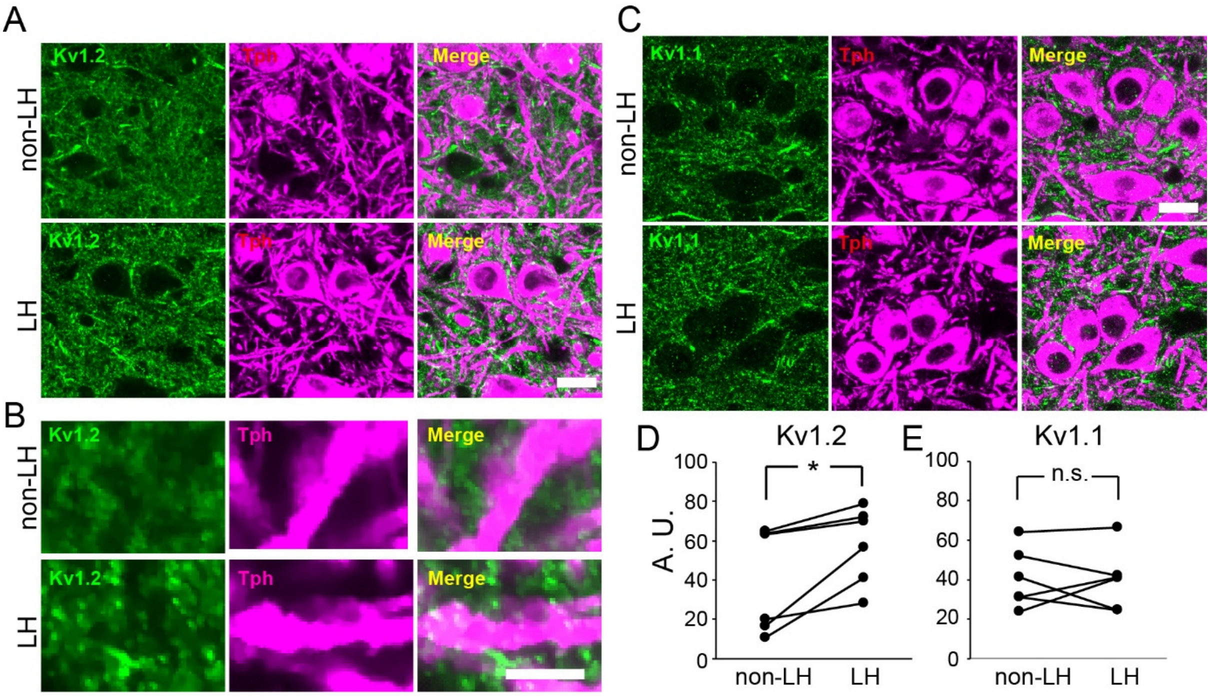

Supplementary Figure 6. Immunofluorescence of Kv1.2 is enhanced in the DRN in LH rats

(**A**) Double immunofluorescence showing Kv1.2 and Tph distributions in non-LH (upper) and LH (lower) rats. Scale bar, 20 μm. (**B**) Higher-magnification images showing Kv1.2 and Tph signals in non-LH (upper) and LH (lower) rats. Scale bar, 5 μm. (**C**) Double immunofluorescence showing Kv1.1 and Tph distributions in non-LH (upper) and LH (lower) rats. Scale bar, 20 μm. Similar images shown in A-C were obtained for three non-LH and three LH rats. (**D, E**) Mean fluorescent intensities per pixel of Kv1.2 (D) or Kv1.1 (E) in non-LH and LH rats. Data were obtained from six DRN images from three non-LH and six DRN images from three LH rats. Data obtained using the same imaging conditions are connected with lines. *, $p < 0.05$, paired t-test.

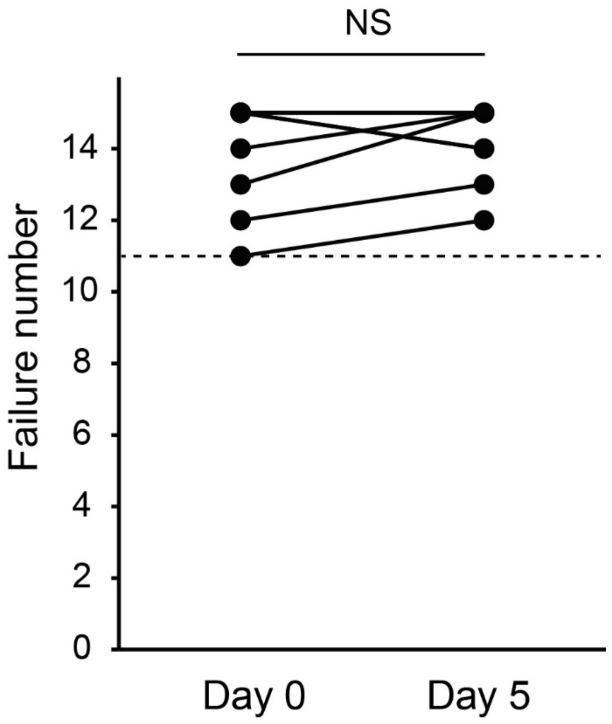

Supplementary Figure 7. LH persists for 5 days after the AT session

Failure numbers at day 0 and day 5 after the AT session (rats = 10). The broken line represents the threshold of LH. There was no statistical significance (*df*=9, *p*=0.343, paired *t*-test).

Supplementary Table 1. Individual data in Table 1.

naïve			non-LH			LH			naïve+DTX			non-LH+DTX			LH+DTX		
RMP	R_{input}	τ	RMP	R_{input}	τ	RMP	R_{input}	τ	RMP	R_{input}	τ	RMP	R_{input}	τ	RMP	R_{input}	τ
(mV)	(MΩ)	(ms)	(mV)	(MΩ)	(ms)	(mV)	(MΩ)	(ms)	(mV)	(MΩ)	(ms)	(mV)	(MΩ)	(ms)	(mV)	(MΩ)	(ms)
-51.54	697.02	31.8	-61.55	585.68	46.9	-58.35	376.95	46	-57.56	490.99	22.5	-55.26	733.58	21.698	-50.68	413.72	38.2
-49.39	573.16	30.4	-49.99	451.29	46	-56.56	708.17	55.7	-46.39	331.96	29.1	-60.85	553.61	21.254	-54.12	649.13	59.7
-56.27	293.43	35.5	-49.99	564.22	50.7	-45.21	438.82	43.7	-39.52	522.2	46.7	-59.45	675.59	26.488	-53.26	603.8	38.2
-43.38	425.9	38.1	-52.98	585.8	46.3	-38.63	509.24	48.1	-48.96	400.4	36.3	-53.26	533.18	40.4	-49.82	431.19	33.8
-50.68	563.39	33.6	-45.81	540.14	43.5	-48.2	552.49	42.6	-54.98	507.78	36.8	-59.27	513.47	35	-49.82	370.36	37.7
-48.54	496.83	37.7	-54.47	326.96	36.9	-56.56	686.57	59.6	-60.13	223	29.8	-54.98	376.36	32.5	-56.7	506.76	57.2
-45.53	311.96	39.6	-65.53	531.56	31	-53.28	619.97	39.3	-61.85	422.5	48.6	-60.13	385.13	38.5	-52.4	726.49	53.9
-48.96	485.78	31.1	-55.96	518.15	44	-54.12	505.58	54							-59.27	668.73	40.7
-64.43	591.66	49.8	-50.29	408.58	37.6	-50.9	490.41	35.4							-60.13	709.25	42.9
-58.84	478.13	56.4	-38.66	472.81	45.3	-57.56	383.69	41.7							-33.93	479.62	24.1
-64.43	699.48	53.7	-63.57	509.41	61.1	-51.54	332.22	37							-51.97	396.78	26
-55.84	739.05	63.4	-60.99	380.28	49.3	-54.98	465.65	71.8							-58.41	576.5	53.9
-60.13	424.14	45.7	-55.84	556.19	41.1	-54.12	448.83	58.9							-54.12	575.34	41.9
-56.7	400.4	61.7	-53.26	475.18	63.5	-59.7	563.05	49.6									
-60.99	422.04	41.3	-54.12	534.47	54.1	-46.39	557.58	47.1									
-63.57	563.23	56.8	-54.12	491.25	52.7	-54.98	524.34	30.5									
			-56.68	397.68	24.209	-36.51	528.95	29.8									
			-49.82	553.67	57	-51.54	656.87	53.4									
						-51.54	605.11	26.9									
						-52.4	341.85	34.6									
						-47.25	566.93	23.4									
						-41.23	441.44	40.8									
						-60.36	538.96	27.49									
						-58.41	590.71	49.4									
						-59.27	496.48	33.9									

RMP, resting membrane potential; R_{input}, input resistance; τ, time constant of hyperpolarizing potential

Supplementary Table 2. Individual data in Table 2.

naïve				non-LH				LH			
Amplitude (mV)	Half-width (ms)	Threshold (mV)	AHP amp (mV)	Amplitude (mV)	Half-width (ms)	Threshold (mV)	AHP amp (mV)	Amplitude (mV)	Half-width (ms)	Threshold (mV)	AHP amp (mV)
79.346	1.05	-34.851	-23.041	97.381	1.3	-37.445	-29.938	83.07	1.25	-36.011	-21.697
79.163	0.95	-31.006	-21.392	89.203	0.85	-32.959	-24.444	86.791	1.65	-29.083	-33.936
89.172	1.05	-33.356	-32.837	86.639	0.95	-36.804	-25.299	83.923	1.45	-40.283	-19.501
81.482	1.05	-36.194	-22.674	92.072	0.95	-32.593	-28.045	75.897	1.3	-33.142	-27.13
76.477	1.05	-30.823	-33.752	90.638	1	-34.607	-27.191	93.506	1	-29.846	-33.539
83.405	0.95	-35.675	-25.39	83.924	1.15	-23.529	-43.579	92.804	1.05	-32.776	-32.898
79.071	0.95	-36.072	-24.323	98.878	1.2	-40.894	-29.815	90.363	1.15	-35.004	-31.372
89.843	0.8	-36.407	-27.1	87.219	0.95	-35.614	-24.475	72.234	1	-24.841	-33.295
84.565	0.9	-38.849	-20.874	88.043	0.9	-29.236	-26.703	82.062	0.95	-33.875	-24.749
84.228	0.85	-36.163	-24.659	90.698	0.95	-32.837	-26.917	74.279	0.85	-32.745	-28.87
85.694	1	-36.133	-26.397	71.29	1.3	-28.809	-30.364	90.576	0.9	-40.985	-24.384
87.341	1.05	-39.459	-25.33	90.608	0.8	-35.584	-29.693	86.7	0.85	-34.119	-23.682
75.135	0.9	-29.053	-30.059	86.242	0.85	-34.454	-22.583	81.756	1.1	-34.271	-22.309
80.2	0.8	-37.689	-23.987	82.671	0.9	-32.104	-33.753	83.648	0.95	-30.426	-33.844
82.611	0.9	-35.645	-27.252	89.904	0.95	-30.914	-29.145	80.78	0.95	-35.431	-27.435
82.916	1.05	-35.95	-29.815	89.325	0.95	-31.494	-34.729	92.437	1.1	-36.377	-30.121
				84.412	0.9	-33.661	-34.606	84.686	1	-39.825	-23.041
				83.465	1.45	-34.973	-24.14	85.51	0.95	-37.811	-22.736
								86.823	1.05	-33.203	-31.097
								85.143	0.85	-34.271	-29.023
								77.942	0.95	-33.783	-24.536
								72.357	1	-29.602	-29.663
								79.163	1.4	-33.539	-27.466
								86.762	0.95	-39.551	-27.13
								92.163	0.75	-39.825	-28.717

AHP, afterhyperpolarization
